# Parkin Precipitates on Mitochondria via Aggregation and Autoubiquitination

**DOI:** 10.3390/ijms24109027

**Published:** 2023-05-19

**Authors:** Mustafa T. Ardah, Nada Radwan, Engila Khan, Tohru Kitada, M Emdadul Haque

**Affiliations:** 1 Department of Biochemistry and Molecular Biology, College of Medicine and Health Sciences, United Arab Emirates University, Al Ain P.O. Box 15551, United Arab Emirates; 2 Otawa-Kagaku, Parkinson Clinic and Research, Kamakura 247-0061, Japan

**Keywords:** Parkin, Parkinson’s disease, neurodegeneration, ubiquitin

## Abstract

The loss of the E3 ligase Parkin, in a familial form of Parkinson’s disease, is thought to cause the failure of both the polyubiquitination of abnormal mitochondria and the consequent induction of mitophagy, resulting in abnormal mitochondrial accumulation. However, this has not been confirmed in patient autopsy cases or animal models. More recently, the function of Parkin as a redox molecule that directly scavenges hydrogen peroxide has attracted much attention. To determine the role of Parkin as a redox molecule in the mitochondria, we overexpressed various combinations of Parkin, along with its substrates FAF1, PINK1, and ubiquitin in cell culture systems. Here, we observed that the E3 Parkin monomer was surprisingly not recruited to abnormal mitochondria but self-aggregated with or without self-ubiquitination into the inner and outer membranes, becoming insoluble. Parkin overexpression alone generated aggregates without self-ubiquitination, but it activated autophagy. These results suggest that for damaged mitochondria, the polyubiquitination of Parkin substrates on the mitochondria is not indispensable for mitophagy.

## 1. Introduction

While Parkinson’s disease (PD) is a highly frequent, sporadic movement disorder, *parkin*, *PINK1*, and *DJ-1* have been identified as early onset, autosomal recessive PD genes, the loss of function of which causes dopaminergic neurodegeneration in the human midbrain [1,2,3]. Mutations in the *parkin* gene are clinically known as autosomal recessive juvenile Parkinsonism (AR-JP). In 2000, Parkin was recognized as an E3 ubiquitin ligase that ubiquitinates its substrates to be degraded in the proteasome system [4]. In 2010, two independent research groups elucidated that a mitochondrial protein, PINK1, recruits Parkin to damaged mitochondria and activates mitophagy, suggesting that the loss of Parkin cannot ubiquitinate its substrates on the mitochondria, resulting in the accumulation of abnormal mitochondria [5,6]. Since then, numerous studies on Parkin/PINK1-related mitophagy have been reported, in which mitochondrial uncouplers such as carbonyl cyanide m-chlorophenylhydrazone (CCCP) have been used to collapse the mitochondrial membrane’s potential as a model of damaged mitochondria. Often, some tagged Parkin proteins, especially GFP-Parkin, have been prepared to monitor E3 ligase activity [6,7]. It is noteworthy that Parkin has another dominant structural feature that prevents oxidative stress and protects the mitochondria from oxidative damage. Parkin was recently shown to be an excellent redox molecule that reacts directly with and instantaneously eliminates hydrogen peroxide (H_2_O_2_) [8,9,10,11]. Thus, the two major dysfunctions of the Parkin protein seem to configure the profound intracellular pathological state in AR-JP brains. However, it has never been considered that, via mitochondrial depolarization by CCCP, a significant amount of H_2_O_2_ leaks from the affected mitochondria into the cytosol in a cell and cytosolic Parkin participates in the elimination of the mitochondrial H_2_O_2_ before the E3 ligase Parkin begins an enzymatic reaction. Considering this, the purpose of our current study was to explore and integrate the complicated cellular pathology caused by the two main dysfunctions of the Parkin protein. To monitor the primary proteins’ actions amid Parkin-related mitophagy under oxidative stress in cell culture, we overexpressed different combinations of tagged or untagged Parkin (HA-Parkin and GFP-Parkin), HA-ubiquitin, FLAG-FAF1 (Parkin’s substrate), and FLAG-PINK1 in cells and treated them with one of three oxidative stress agents: CCCP, an uncoupler of mitochondrial membranes; H_2_O_2_, which causes exogenous oxidative stress outside mitochondria; or 1-Methyl-4-phenyl-1,2,3,6-tetrahydropyridine (MPTP/MPP^+)^, which causes oxidative stress inside mitochondria. In the current study, we observed structural and dynamic changes in each protein following treatment. The experiments allowed us to assess the validity of using CCCP, tagged Parkin (especially large tags), and PINK1 function in the current AR-JP cellular model. Our unique experimental approach will also provide new insights into AR-JP pathogenesis and help to develop cause-directed therapeutics for AR-JP.

## 2. Results

### 2.1. Characterization of the Basic Properties of the Redox Molecule, PARKIN and Its Substrate FAF1

**Figure 1:**
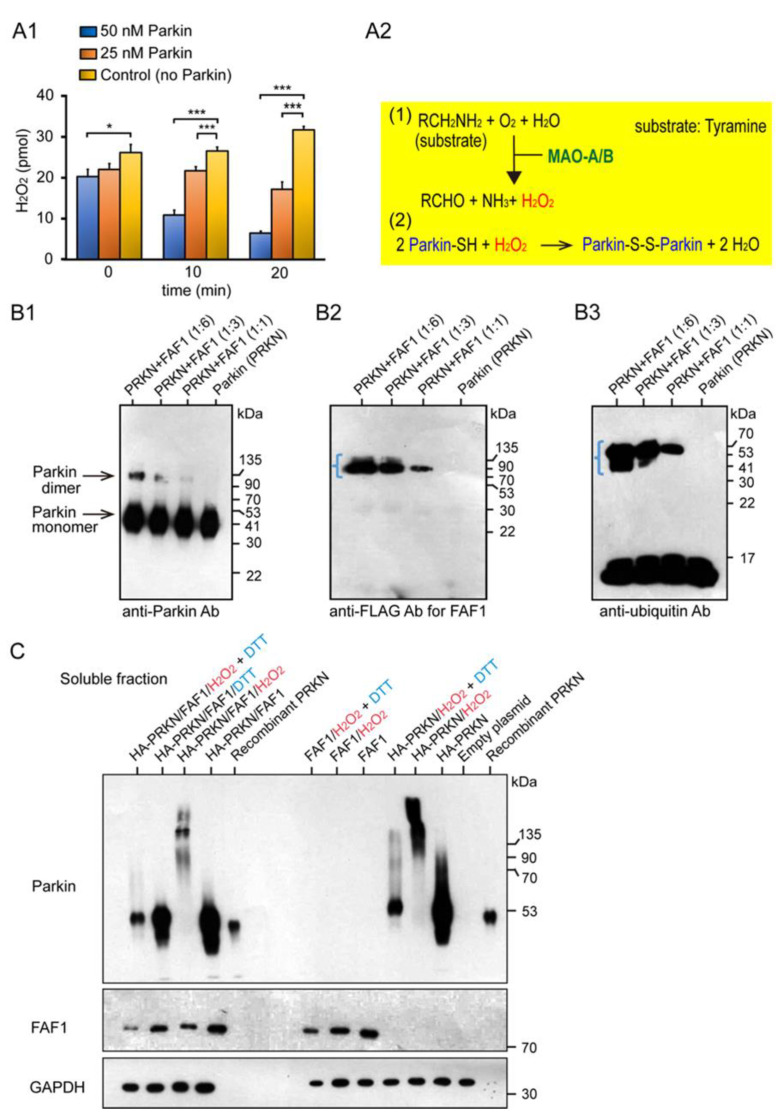
Characterization of the basic properties of the redox molecule Parkin and its substrate FAF1. (**A1**): MAO Assay: Parkin reacts with and reduces H_2_O_2_, a by-product of the chemical reaction between MAO-A and tyramine. Error bars represent the mean ± SD values of three experiments; statistical analysis was calculated using one-way ANOVA, followed by the Bonferroni Multiple Comparison test. *** *p* < 0.0001; * *p* < 0.05. (**A2**): Chemical reaction equations of MAO with tyramine and of Parkin protein with H_2_O_2_. (**B**): WB for FAF1 Polyubiquitination Assay using the Parkin E3 ligase; E3 activity does not allow autoubiquitination. (**B1**): WB analysis using anti-Parkin antibodies. (**B2**): WB analysis using anti-FLAG antibodies for FAF1. (**B3**): WB analysis using anti-Ubi antibodies. The same WB membrane was used for all of (**B1**–**B3**). (**C**): Overexpression of Parkin/FAF1 in HEK293 (HEK) cells with/without H_2_O_2_: WB for soluble fractions. 20 mM H_2_O_2_ or 100 mM DTT were exposed for 30 min at room temperature. DTT was added 5 min before collecting the cells.

We characterized the basic properties of the redox molecule Parkin in the mitochondria and its substrate FAF1. Monoamine oxidase (MAO) is present in the mitochondria of the substantia nigra (SN) and locus coeruleus (LC). In the MAO assay, the MAO-A degraded its substrate, tyramine, and produced H_2_O_2_ as a by-product (Figure 1A1,A2). When the Parkin protein was added to this reaction system, it was expected to react with and reduce H_2_O_2_. In fact, two concentrations of Parkin (i.e., final 25 nM and 50 nM) were added in contrast to the control group, and H_2_O_2_ was measured over time (i.e., 0 min, 10 min, 20 min). Compared with the control group, the Parkin-added groups showed more significant H_2_O_2_ reduction over time, at 0 min (50 nM Parkin), at 10 min (25 nM and 50 nM Parkin), and at 20 min (25 nM and 50 nM Parkin).

Sul et al. reported that FAF1 is a substrate of Parkin [12]. We used the Parkin protein, supplied by R&D Systems, to perform the FAF1 polyubiquitination assay. In this reaction system, the Parkin is adjusted to have “negligible” E3 ligase activity, as determined by the lack of autoubiquitination in the assay. Following the termination of the FAF1 polyubiquitination assay, Western blot (WB) analyses were performed for these samples using anti-Parkin, anti-FLAG for FAF1, and anti-ubiquitin (Ubi) antibodies. The WB analysis using anti-Parkin antibodies showed two strong, high-intensity bands (Figure 1B1), which indicated a Parkin monomer and its dimer, as this type of Parkin is not self-ubiquitinated (autoubiquitinated). FAF1 seemed to be ubiquitinated with a small ubiquitin chain, which was confirmed by the WB using anti-Ubi antibodies, indicating a short rectangular band around the FAF1 molecular weight size (blue angle bracket in Figure 1B2,B3). Next, we overexpressed the HA-Parkin and FLAG-FAF1 in HEK293 (HEK) cell cultures to investigate whether the Parkin ubiquitinated the FAF1 protein at the cell culture level. After collecting cell lysates of HEK cells with the two overexpressed genes and separating them into soluble and insoluble fractions, we performed WB analyses for the two. While mitochondrial oxidative stress (20 mM H_2_O_2_) facilitated the generation of a longitudinal smear, including high-molecular-weight (HMW) Parkin aggregates, the antioxidant (100 mM DTT) changed the smear into a small longitudinal smear in the WB from the soluble fraction using the anti-Parkin antibodies (Figure 1C; left side). It is unclear whether FAF1 is polyubiquitinated. Similar results were obtained when only the Parkin was overexpressed (Figure 1C; right side).

### 2.2. Overexpression of Three Genes—HA- or GFP-Tagged Parkin/FLAG-FAF1/HA-Ubi—In HEK Cell System and Exposure to H_2_O_2_ or CCCP

**Figure 2:**
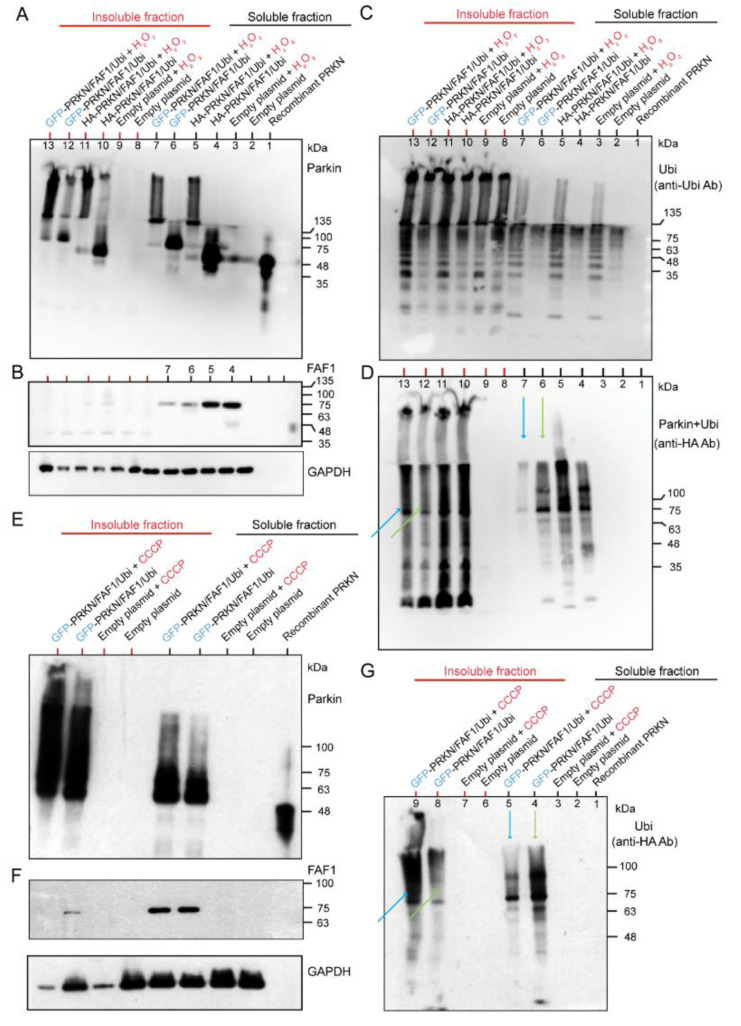
Hydrogen peroxide (H_2_O_2_) exposure to HEK cells transfected with GFP-Parkin or HA-Parkin, FAF1, and Ubi. Twenty-four hours after transfections, HEK cells were treated with H_2_O_2_ (20 mM, 30 min), and separated into Triton X-100 soluble and insoluble fractions. (**A**): WB for soluble and insoluble fractions using an anti-Parkin antibody. (**B**): WB for soluble and insoluble fractions using anti-FLAG antibodies for FAF1. (**C**): WB for soluble and insoluble fractions using anti-Ubi antibodies. (**D**): WB for soluble and insoluble fractions using anti-HA antibodies for Ubi and HA-Parkin. The same WB membrane was used for all of (**A**–**D**). CCCP exposure to HEK cells were transfected with GFP-Parkin, FAF1, and Ubi. Twenty-four hours after transfection, the cells were treated with CCCP (20 μM, 6 h) and then separated into triton X-100 soluble and insoluble fractions. (**E**): WB for soluble and insoluble fractions using anti-Parkin antibodies. (**F**): WB for soluble and insoluble fractions using anti-FLAG antibodies for FAF1. (**G**): WB for soluble and insoluble fractions using anti-HA antibodies for Ubi. The same WB membrane was used for all the figures in (**E**–**G**).

We have demonstrated that the Parkin ubiquitinated FAF1 at the biochemical reaction level (Figure 1B). However, while Parkin and FAF1 were overexpressed at the cell culture level, the polyubiquitination of the FAF1 was not evident (Figure 1C). To investigate the contribution of the ubiquitination of the Parkin to its substrates (FAF1) and its autoubiquitination by expressing the ubiquitin under H_2_O_2_ treatment, and to study the effect of the different tags of the Parkin protein, we overexpressed three genes—HA- or GFP-tagged Parkin/FLAG-FAF1/HA-Ubi—in HEK cell lines. After collecting the cell lysates of the HEK cells with the three overexpressed genes, we separated them into soluble and insoluble fractions of the lysates and performed WB analyses for the two fractions. The WB analysis using the anti-Parkin antibodies showed that the H_2_O_2_ facilitated the generation of longitudinal HMW smear bands compared to the controls (without H_2_O_2_), in both HA-Parkin- and GFP-Parkin-overexpressing HEK cells (Figure 2A). This tendency was observed to a greater extent in the insoluble fraction than in the soluble fraction (Figure 2A). Thus, the H_2_O_2_ led to the generation of the insoluble Parkin aggregates. The FAF1 derived from the FAF1 gene vector was stained with anti-FLAG antibodies. In the WB, the FAF1 protein showed a single band, or, at most, a very thin band, attached to a small ubiquitin chain only in the soluble fraction (Figure 2B). These results suggest that a longitudinal band of polyubiquitination in the WB mainly constitutes the Parkin’s autoubiquitination rather than the FAF1′s polyubiquitination (Figure 2B,C). When blotting the same membrane using the anti-Ubi antibodies, the H_2_O_2_ facilitated the generation of a long HMW smear (Figure 2C), which was clearer in the soluble fraction than in the insoluble fraction (Figure 2C). Thus, the polyubiquitination of proteins (mainly Parkin) is enhanced under oxidative stress. The anti-HA antibodies reacted with the HA-Parkin and HA-ubiquitin. In Figure 2D, the effect of the H_2_O_2_ on the polyubiquitination of the GFP-Parkin is much more intense in the insoluble fraction than in the soluble fraction (lane 7 in the soluble fraction and lane 13 in the insoluble fraction in Figure 2D; blue arrows). In contrast, without H_2_O_2_ treatment, the polyubiquitination of the GFP-Parkin was more intense in the soluble fraction than in the insoluble fraction (lane 6 in the soluble fraction and lane 12 in the insoluble fraction; Figure 2D; yellow-green arrows). This suggests that GFP-Parkin can be aggregated more easily using H_2_O_2_ treatment, and as a result, becomes insoluble. A similar result was obtained when CCCP was used instead of H_2_O_2_ (Figure 2G).

A WB analysis using anti-Parkin antibodies showed that the CCCP facilitated the generation of longitudinal HMW smears compared to controls (without CCCP) in the GFP-Parkin/FLAG-FAF1/HA-Ubi overexpressing cell cultures (Figure 2E). This tendency was observed to a greater degree in the insoluble fraction than in the soluble fraction (Figure 2E). Thus, the CCCP led to the generation of insoluble Parkin aggregates. FAF1 derived from the FAF1 gene vector was probed with anti-FLAG antibodies. In the WB, the FAF1 protein showed a single band, mainly in the soluble fraction of cells with/without CCCP (Figure 2F). These results suggest that a longitudinal band of polyubiquitination in the WB is mainly composed of the Parkin’s autoubiquitination rather than the FAF1 polyubiquitination (Figure 2F,G). The WB analysis using anti-HA antibodies, which react with HA-ubiquitin, also implies that the CCCP induces the aggregation of the insoluble polyubiquitinated HMW Parkin (Figure 2G). In Figure 2G, the effect of the CCCP on the polyubiquitination of the GFP-Parkin is much more intense in the insoluble fraction than in the soluble fraction (lane 5 in the soluble fraction and lane 9 in the insoluble fraction in Figure 2G; blue arrows). In contrast, without CCCP treatment, the polyubiquitination of the GFP-Parkin was more intense in the soluble fraction than in the insoluble fraction (lane 4 in the soluble fraction and lane 8 in the insoluble fraction; Figure 2G; yellow-green arrows). This suggests that GFP-Parkin can be aggregated more easily using CCCP treatment, and, as a result, becomes insoluble. As mentioned in Figure 2D, a similar result was obtained in the same experiment using H_2_O_2_ instead of CCCP (Figure 2D). In conclusion, CCCP treatment of cell cultures overexpressing GFP-Parkin/FLAG-FAF1/HA-Ubi genes demonstrated the same result as that of the H_2_O_2_ treatment for the same cell cultures (Figure 2A–G). Both chemicals facilitated Parkin aggregation and polyubiquitination rather than FAF1 polyubiquitination. Polyubiquitinated Parkin aggregates are highly insoluble and easily precipitate.

#### 2.2.1. Effects of H_2_O_2_ and CCCP Treatment on Different Fractions of HEK Cell System Overexpressing HA-Tagged or Untagged Parkin/FAF1 or Untagged Parkin Alone

**Figure 3:**
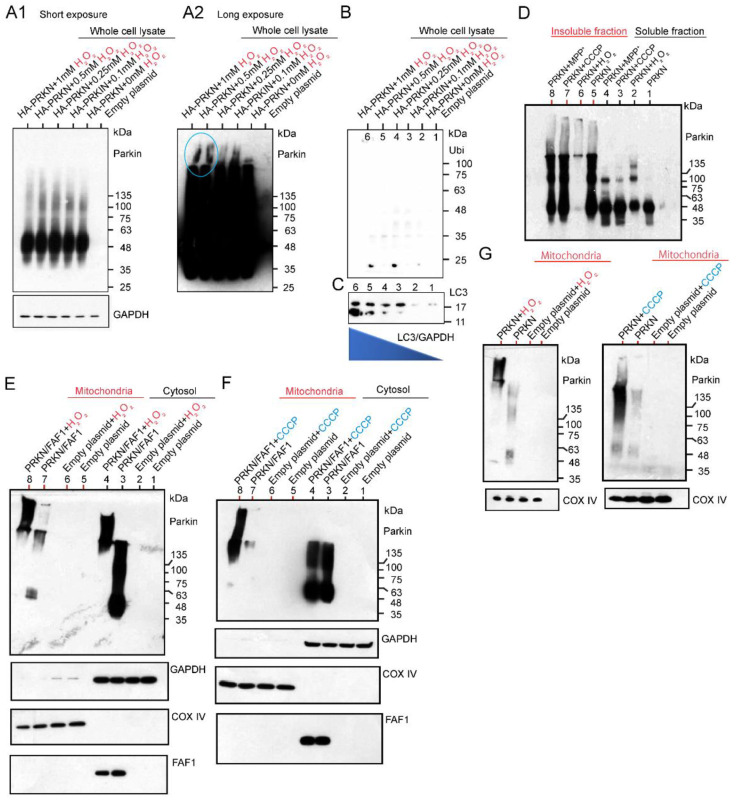
Exposure of HA-Parkin over-expressed HEK cells with different concentrations of H_2_O_2_. Twenty-four hours after transfection, HEK cells were treated with H_2_O_2_, and whole cell lysates (WCL) were prepared and utilized for WB analysis. (**A1**,**A2**): WB for WCL using an anti-Parkin antibody. WB results were evaluated at different exposure times. (**B**): WB for WC using an anti-Ubi antibodies. (**C**): WB for WCL using anti-LC3 antibodies. The same WB membrane was used for all of (**A**–**C**). H_2_O_2_ (20 mM) and CCCP (20 μM) effect in Triton X-100 soluble and insoluble fractions from HEK cells overexpressing untagged Parkin alone. (**D**): WB for soluble and insoluble fractions using anti-Parkin antibodies under non-reducing conditions. H_2_O_2_ vs. CCCP effect in mitochondrial and cytosolic fractions from HEK cells overexpressing untagged Parkin/FAF1 (**E**,**F**) or untagged Parkin alone (**G**).

After exposing HEK cells overexpressing HA-Parkin alone to various concentrations of H_2_O_2_, whole cell lysates (WCL) were extracted, and then the WB was performed using anti-Parkin antibodies. This result indicates that a lower dose of H_2_O_2_ leads to high-intensity smears, which are ~135 kDa in size, in the WCL (Figure 3A1). However, the WB results after long exposure using the same membrane showed that the HMW of a large smear was observed in the lanes loaded with samples treated with a higher dose of H_2_O_2_ (Figure 3A2; blue-colored circles). Thus, a higher dose of H_2_O_2_ generated the HMW of Parkin aggregates. The WB analysis using anti-Ubi antibodies did not detect any smears. This result suggests that the longitudinal smear, which was detected by the anti-Parkin antibodies, is composed of Parkin aggregates without polyubiquitin chains (Figure 3B). Through the WB analysis of the same membrane using anti-LC3 antibodies, we assessed the autophagy activity in the HEK cells overexpressing HA-Parkin under H_2_O_2_ treatment. Interestingly, LC3 expression had a higher intensity of strong Parkin aggregation when treated with a higher dose of H_2_O_2_. This suggests that even simple Parkin aggregation without polyubiquitin chains induces autophagy (Figure 3C), although H_2_O_2_ would also affect the aggregation or other changes of other proteins. However, additional experiments confirmed that the concentration of the H_2_O_2_ contributed the most to the enhancement of the LC3, but may be modulated by the Parkin expression (Supplementary Figures S1 and S2).

Next, we examined the effects of CCCP and H_2_O_2_ on Parkin aggregation by expressing the Parkin in the HEK cells and adding the three chemicals after 24 h, harvesting the cells after exposure to different toxins, and separating them into soluble and insoluble fractions for the WB under non-reducing conditions (Figure 3D). Under the non-reducing conditions, the WB was performed without boiling the sample and without using loading dye containing reducing agents such as dithiothreitol (DTT). This is because Parkin aggregates are degraded by reducing agents and partially converted to E3-active monomers. We also decided to use non-tagged Parkin to eliminate the unexpected effect of tags (especially GFP-tag). The WB results showed that all the chemicals generated various insoluble polymers of Parkin, and in the soluble fraction, they mainly generated monomers and dimers of Parkin. Strikingly, in the insoluble fraction, high concentrations of H_2_O_2_ seemed to cause Parkin to form HMW aggregates that were narrow in size (lane 6 in Figure 3D). We investigated the effect of H_2_O_2_ and CCCP on Parkin protein dynamics in the mitochondrial and cytosolic fractions of cell cultures overexpressing Parkin alone or Parkin/FAF1 together. The WB analysis using anti-Parkin antibodies revealed that 20 mM H_2_O_2_ treatment generated HMW-smeared bands both in the mitochondrial fraction and the cytosolic fraction of the cell cultures with Parkin/FAF1 overexpression compared to the control (Figure 3E,F). These results suggest that polyubiquitinated Parkin aggregations deposit in the mitochondria as well as in the cytosol, especially under oxidative stress. Next, we overexpressed the Parkin alone in the cell cultures and obtained mitochondrial and cytosolic fractions. The WB analysis using anti-Parkin antibodies revealed that, similar to Parkin/FAF1 overexpression, 20 mM H_2_O_2_ treatment generated HMW-smeared bands both in the mitochondrial fraction (Figure 3G) and the cytosolic fraction () of the cell cultures with Parkin overexpression and 20 μM CCCP treatment (Figure 3G). This result suggests that Parkin deposits as high-molecular-weight aggregates in the mitochondria, especially under oxidative stress. In conclusion, the WB analysis using the anti-parkin antibodies showed that both H_2_O_2_ and CCCP treatments induced intense and HMW-smeared bands compared to each control. This result suggests that polyubiquitinated and aggregated Parkin deposits in the mitochondria rather than the translocation of Parkin to the mitochondria. This is because the Parkin monomer alone has E3 ligase activity but is absent or very pale in the WB analysis (Figure 3E–G). Changes in the polyubiquitination of the substrate FAF1 were hardly observed, and no remarkable difference was observed when compared to the control group (non-chemical-treated group; Figure 3E,F).

#### 2.2.2. Investigation of Aggregation and Insolubility of Parkin Protein Using MPTP-Treated PD Mouse Model

It is well known that the administration of the neurotoxin MPTP to experimental animals causes PD-like symptoms and the degeneration of the substantia nigra [13]. MPTP is taken into glial cells and metabolized to MPP^+^, which is then taken up by dopaminergic neurons via the dopamine transporter. MPP^+^ selectively inhibits the mitochondrial respiratory chain complex I, resulting in mitochondrial failure [13]. As shown in Figure 3D, MPTP caused Parkin aggregates and insolubility at the cell culture level. Thus, as an additional experiment, we validated these results using MPTP-treated mice, which are often used as PD animal models.

**Figure 4:**
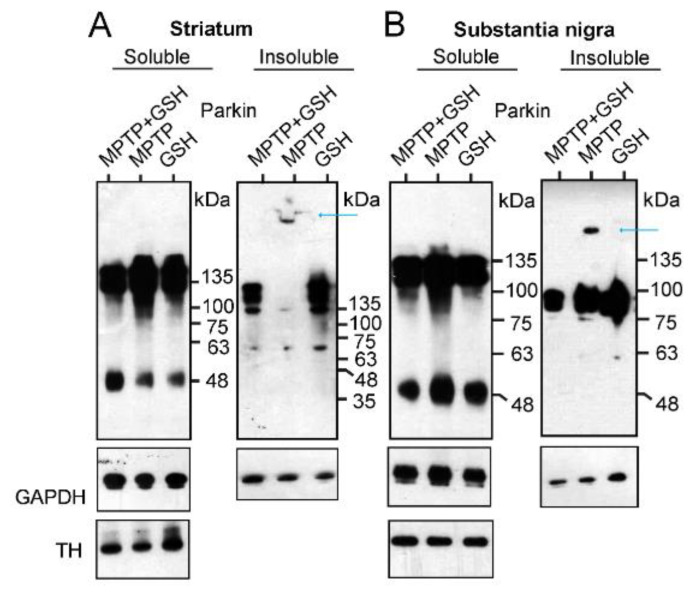
Mice received intraperitoneal injections of MPTP, GSH, and MPTP + GSH as mentioned in the method section. Tissues were collected from the striatum (**A**) or substantia nigra (**B**) and separated into Triton X-100 soluble and insoluble fractions for WB analysis to measure the intensity of Parkin dimer and GAPDH, and their ratios (dimer: GAPDH).

After treating the mice with 1-methyl-4-phenyl-1,2,3,6-tetrahydropyridine (MPTP), glutathione (GSH), or MPTP plus GSH, tissue samples from the striatum and substantia nigra were collected and separated into Triton X-100 soluble and insoluble fractions for the WB analysis. In the WB of the soluble fractions of the striatum (Figure 4A) and substantia nigra (Figure 4B), the ratio of intensity of the Parkin dimer to GAPDH (dimer: GAPDH) in MPTP-treated mouse brains was higher than that in mice treated with either GSH or MPTP plus GSH {MPTP plus GSH: MPTP: GSH = 1.0:1.8:1.1 in the striatum; 1.0:1.2:1.1 in the substantia nigra (analyzed by Image J, NIH, USA)}. Interestingly, in the WB of the insoluble fractions of the striatum and substantia nigra in MPTP-treated mouse brains, an extreme HMW of Parkin aggregations was detected compared to those of Parkin oligomers (blue arrows in Figure 4A,B). In particular, some MPTP-treated mouse striata were stuck in the comb space of the gel (Figure 4A).

Thus, MPTP selectively inhibits the activity of complex I in the mitochondria of dopamine neurons and leaks ROS from the mitochondria. In turn, Parkin neutralizes ROS and transforms its monomers and oligomers into an extreme HMW of its aggregation, which is insoluble and precipitates a tripeptide protein (L-γ-glutamyl-L-cysteinyl-glycine), GSH, that is protected against the formation of Parkin aggregates via an antioxidant effect that directly neutralizes ROS with the thiol groups of its cysteine residues.

### 2.3. WB Analysis of Mitochondrial Subfractions after H_2_O_2_ or CCCP Exposure to Cultured Cells Overexpressing Untagged Parkin + FLAG-PINK1, Which Was Performed under Reducing or Non-Reducing Conditions

**Figure 5:**
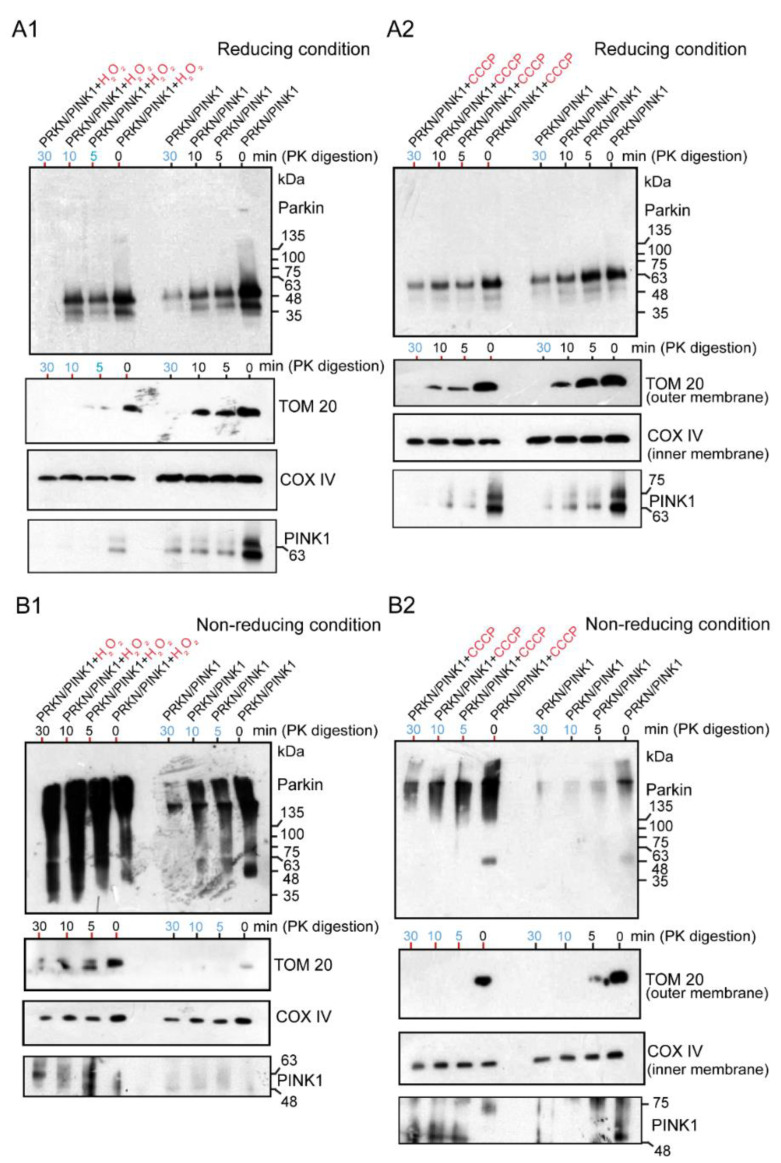
HEK cells were transfected with untagged Parkin and FLAG-PINK1. After 24 h, cells were treated with CCCP (20 μM for 6 h) or H_2_O_2_ (20 mM for 30 min), then were collected and processed for mitochondria isolation. Samples of the mitochondrial fraction were treated with proteinase-K over time (0, 5, 10, 30 min) and finally utilized for WB analysis using an anti-Parkin antibody or other antibodies (TOM20, COX IV, and PINK1). The blue numerals indicate the presence of only the inner membrane after degradation of the outer membrane. (**A1**): the samples (H_2_O_2_-treated Parkin) were loaded under the reducing condition. (**A2**): the samples (CCCP-treated Parkin) were loaded under the reducing condition. (**B1**): the samples (H_2_O_2_-treated Parkin) ware loaded under the non-reducing condition (no boiling and no reducing agent in loading dye). (**B2**): the samples (CCCP-treated Parkin) were loaded under the non-reducing condition.

**Figure 6:**
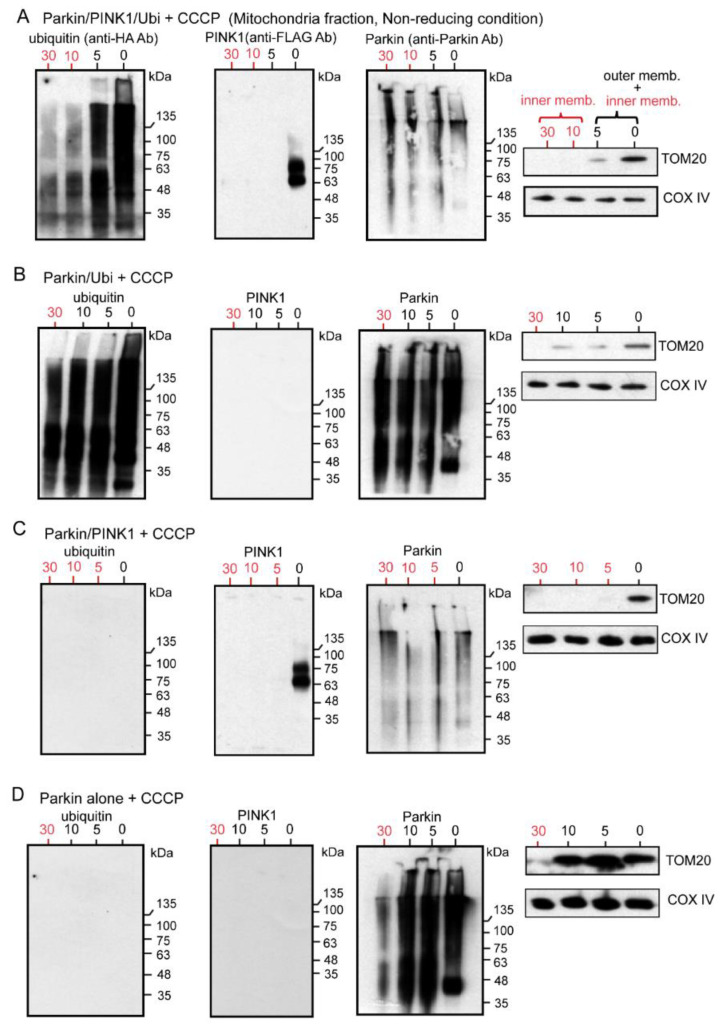
HEK cells were transfected with various combinations of non-tagged Parkin, FLAG-PINK1, and HA-Ubi. After 24 h, cells were treated with CCCP (20 μM for 6 h), then were collected and processed for mitochondria isolation. Samples of the mitochondrial fraction were treated with proteinase-K over time (0, 5, 10, 30 min) and finally utilized for WB analysis under the non-reducing condition. (**A**): Samples derived from HEK cells with Parkin/PINK1/Ubi overexpression. (**B**): Samples derived from HEK cells with Parkin/Ubi overexpression. (**C**): Samples derived from HEK cells with Parkin/PINK1 overexpression. (**D**): Samples derived from HEK cells with Parkin alone overexpression. The red numerals indicate the presence of only the inner membrane after degradation of the outer membrane.

Under the reducing conditions, both the H_2_O_2_-treated (Figure 5A1) and CCCP-treated Parkin (Figure 5A2) were apparently present as monomers, which was interpreted as the recruitment of the E3 Parkin to the mitochondria. The degradation of these mitochondrial samples with Proteinase-K over time showed that the Parkin was present not only in the outer membrane but also in the inner membrane, regardless of the presence or absence of the above chemicals (blue numerals in Figure 5A1,A2). However, to prevent the degradation and monomerization of Parkin aggregates by the reducing agent DTT in a loading dye, HEK cells overexpressing Parkin/PINK1 were treated with H_2_O_2_ (Figure 5B1) or CCCP (Figure 5B2) and the mitochondrial fraction was subjected to WB analysis under non-reducing conditions after Proteinase-K digestion. Surprisingly, continuous longitudinal smears from HMW of size were observed in the oxidative stress (H_2_O_2_ and CCCP) groups, but predominantly included the control groups (Figure 5B1,B2). These Parkin aggregates of various sizes are present not only in the outer membrane, as pointed out by known hypotheses, but also non-specifically in the inner membrane (blue numerals in Figure 5B1,B2). Notably, the experiment in Figure 5B2 partially overlapped with the experiment in Figure 6C. PINK1 was strongly observed in the outer membrane of the mitochondria (Figure 5A1–B2 and Figure 6C).

### 2.4. WB Analysis of Mitochondrial Subfractions after CCCP Exposure to Cultured Cells Overexpressing Various Combinations of Un-Tagged Parkin, FLAG-PINK1, and HA-Ubi, Which Was Performed under Non-Reducing Condition

The following points were noted from the results of our series of experiments. First, the WB was performed under non-reducing conditions to observe the original state of Parkin. To eliminate the effect of tags on the Parkin, we overexpressed the Parkin without tags. The Parkin, PINK1, and Ubi were overexpressed in HEK cells in various combinations, and the mitochondrial fractions were extracted after CCCP treatment. These mitochondrial samples were degraded with Proteinase-K over time (0, 5, 10, and 30 min), and each was used for the WB under non-reducing conditions. In all the WB with anti-Parkin antibodies (Figure 6A–D), the first lane labeled “0” showed a continuous smear from the monomer level to the HMW level. Interestingly, the entire smear was thinner in the samples with simultaneous PINK1 expression compared to the sample without PINK1 overexpression (compare Figure 6A,C with Figure 6B,D). This is possibly because PINK1 phosphorylates Parkin, and as a result, this phosphorylation prevents the recognition and binding by anti-Parkin antibodies. Another discovery is that the Parkin is not only observed in the outer membrane but also in the inner membrane (red numerals in Figure 6A–D). Corresponding to this finding, polyubiquitin chains were also observed as a continuous longitudinal smear, ranging from low to high molecular weights on both the outer and inner membranes, which is also a new finding (Figure 6A,B). In contrast, PINK1 was present on the outer membrane after CCCP treatment, as previously reported (Figure 6A,C).

### 2.5. Fluorescent Immunocytochemistry (ICC) to Analyze Accumulation of Parkin in Mitochondria of Cultured Cells Overexpressing Several Combinations of GFP-Parkin, HA-Ubi, and myc-PINK1 after Exposure to CCCP

**Figure 7:**
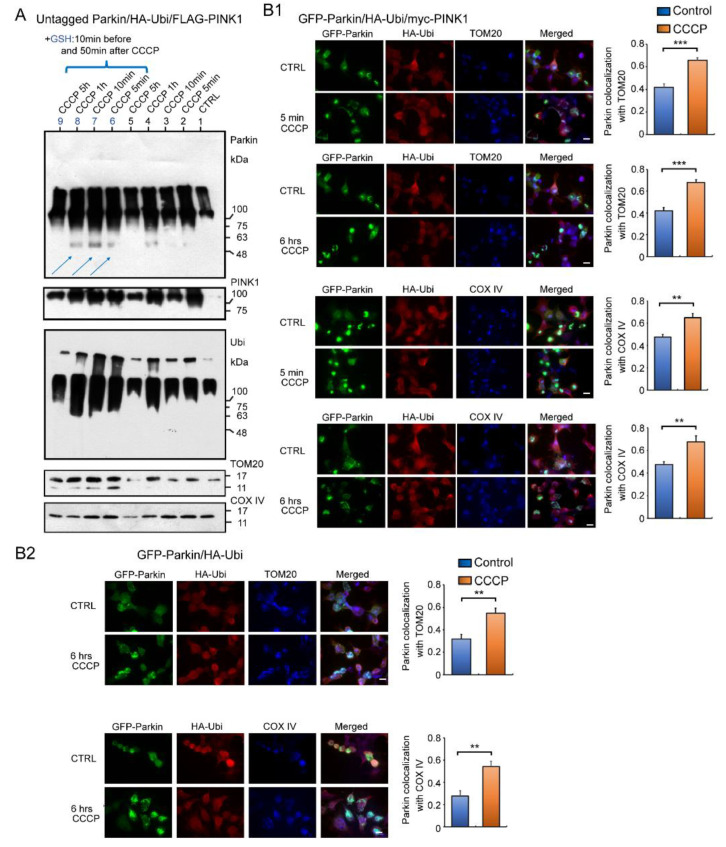
Western blot and fluorescent immunocytochemistry (ICC) of HEK cells overexpressing several combinations of plasmids. (**A**): HEK cells overexpressing untagged Parkin, HA-Ubi, and FLAG-PINK1 were treated with CCCP (20 μM) and collected at specific time intervals (5 min, 10 min, 1 h, 5 h) for WB analysis of mitochondrial fractions. WB analysis using anti-Parkin and other antibodies (for TOM20, COX IV, FLAG-PINK1, and HA-Ubi). Some samples were treated with GSH 10 min before and 50 min after CCCP administration (blue arrows). (**B1**): fluorescent ICC of HEK cells overexpressing GFP-Parkin, HA-Ubi, and myc-PINK1 and fixed 5 min and 6 h after CCCP administration. Anti-HA, anti-COX IV, and anti-TOM20 antibodies were used as primary antibodies. Graphs represent Parkin colocalization with a mitochondrial marker. (**B2**): Fluorescent ICC of HEK cells overexpressing GFP-Parkin and HA-Ubi, and fixed 6 h after CCCP administration. Graphs represent Parkin colocalization with mitochondrial marker. Scale bar 12 μm. *** *p* < 0.0001, ** *p* < 0.001.

If the Parkin reacts to H_2_O_2_ leaked by CCCP and accumulates on mitochondria, it should accumulate rapidly, unlike multistep enzymatic reactions. We therefore administered CCCP to the HEK cells overexpressing untagged Parkin, HA-Ubi, and FLAG-PINK1, and then collected cells at specific time intervals (i.e., five minutes, 10 min, one hour, five hours) for a WB analysis using mitochondrial fractions (Figure 7A). Surprisingly, the WB result obtained after five minutes of CCCP treatment showed that most of the Parkin had already been accumulated in the mitochondria as HMW aggregates and were self-ubiquitinated. Vertical smears were also observed in the non-CCCP-treated samples (control), likely because of a reaction to the small amount of H_2_O_2_ continuously diffusing out of the mitochondria [14]. Interestingly, the addition of GSH, which has a reducing effect in mitochondria, increased the expression of Parkin monomers (blue arrows in Figure 7A).

We also performed an immunofluorescent analysis to confirm the WB result (Figure 7B). The HEK cells were overexpressed with several combinations of GFP-Parkin, HA-Ubi, and myc-PINK1 and treated with CCCP, as indicated in Figure 7B1,B2. A triple-staining analysis using a fluorescence light microscopy revealed overlapping images of the Parkin, Ubi, and mitochondrial markers—both TOM20 and COX IV. In summary, the Parkin aggregates were observed in the mitochondria even in the absence of PINK1. This deposition was also observed in controls without CCCP treatment, although the distribution and concentration of positive mitochondria was relatively mild (See Figure 7A and bar graphs in Figure 7B1,B2). Parkin was present in both the inner and outer mitochondrial membranes. Thus, the immunofluorescence staining data fully support the WB results.

### 2.6. Summary of Results: Functional Model of the Redox Molecule Parkin around Mitochondria

**Figure 8:**
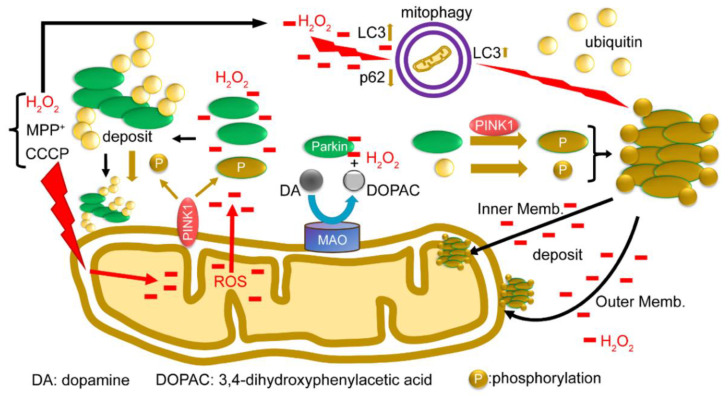
A model for the function of the redox molecule Parkin around mitochondria: When depolarization is triggered in the mitochondrial membrane, all ions, proteins, and ROS in the mitochondria leak or are exposed to the outer membrane. Parkin from the cytoplasm reacts with leaked H_2_O_2_ to aggregate and autoubiquitinate, and non-specifically precipitate into both the inner and outer membrane. PINK1 is exposed to the outer membrane and phosphorylates Parkin and ubiquitin; even when phosphorylated, Parkin does not ubiquitinate the substrate, but rather causes autoubiquitination. Parkin is also presumed to react with H_2_O_2_ generated by MAO-A/B on the outer membrane and to eliminate H_2_O_2_ in dopaminergic neurons. In addition, H_2_O_2_ leaking into the cytoplasm directly stimulates mitophagy (Suppl-Figures S1 and S2). Parkin aggregates also positively modulate mitophagy (Suppl-Figures S1 and S2).

Finally, we present a functional model of the redox molecule Parkin in the mitochondria (Figure 8). When depolarization is triggered in the mitochondrial membrane, all the ions, proteins, and ROS in the mitochondria leak into or are exposed to the outer membrane. Parkin from the cytoplasm reacts with leaked H_2_O_2_ to aggregate and autoubiquitinate and non-specifically precipitates into both the inner and outer membranes. PINK1 is exposed to the outer membrane and phosphorylates Parkin and ubiquitin; however, even when phosphorylated, phosphorylated Parkin does not ubiquitinate the substrate, but rather leads to autoubiquitination and aggregation. Parkin is also presumed to react with H_2_O_2_ generated by MAO-A/B on the outer membrane, and eliminate H_2_O_2_, which may contribute to protecting the substantia nigra from cell death. In addition, H_2_O_2_ leaking into the cytoplasm directly stimulates mitophagy (Suppl-Figures S1 and S2). Parkin aggregates also positively modulate mitophagy (Suppl-Figures S1 and S2). Lastly, we attempted to use the SH-SY5Y cell lines other than HEK cells to characterize the behavior of the endogenous Parkin protein under oxidative stress. We have used SH-SY5Y cells since they express a sufficient level of endogenous Parkin and avoid artifacts that may occur in the experiments due to gene overexpression or cell line specificity. A Western blot analysis of mitochondrial fractions isolated from SH-SY5Y cells under non-reducing conditions showed bands of high-molecular-weight aggregates of endogenous Parkin upon CCCP exposure (Suppl-Figure S3A), which were ubiquitin-positive. By contrast, the same experiment was performed with the same specimens under reducing conditions, but a band of Parkin monomers was detected (Suppl-Figure S3B). WB analysis of this membrane, using an anti-Ubi antibody, showed vertical smears, indicating that the DTT in the loading dye degraded these Parkin aggregates to various ubiquitin-positive sizes.

## 3. Discussion

From a series of current studies, we have identified several critical facts which often essentially conflict with the current dogma: “mitophagy insufficiency due to dysfunctional Parkin/PINK1 pathway.” First, we confirmed that Parkin reacts with and reduces H_2_O_2_ derived from the monoamine oxidase (MAO) enzyme using a MAO activity assay [15]. We also confirmed that FAF1 is a Parkin substrate in vitro (test tube) and at the cell culture level.

Through the multiple gene overexpression system in cell cultures, H_2_O_2_, CCCP, or MPTP treatment caused Parkin self-aggregation and autoubiquitination rather than polyubiquitination of the substrate FAF1. Importantly, our result suggests that WB analysis should be performed under non-reducing conditions because the reducing agent in the loading dye dramatically reduces the polyubiquitinated HMW Parkin aggregates into monomeric parkin. Thus, this hinders the real interpretation of the data.

In the mitochondrial fraction, CCCP (as well as H_2_O_2_) treatment also causes the precipitation of the polyubiquitinated Parkin’s aggregation into the mitochondria, but not that of the Parkin monomer, which only has E3 activity. Parkin overexpression alone causes Parkin aggregation without polyubiquitination, and LC3 is activated as well, suggesting that Parkin aggregation itself positively modulates mitophagy. We also confirmed the self-aggregation and autoubiquitination of the Parkin observed in an MPTP mouse model. Finally, we confirmed that PINK1 seemed to phosphorylate the Parkin, but it did not increase the ligase activity of the Parkin; rather, it promoted its aggregation and autoubiquitination to make it insoluble.

While several groups have reported that Parkin is rapidly recruited to the mitochondria when cells are treated with CCCP, the true molecular mechanism of “rapid recruitment of Parkin to the mitochondria” remains unclear [16,17]. However, our results indicate that the E3 Parkin “monomer” is not recruited to the mitochondria, but its polyubiquitinated aggregates rapidly precipitate into the mitochondria. It has also been reported that Parkin selectively localizes to the outer membrane of the mitochondria, whose membrane potential had been lost, because Parkin polyubiquitinates several types of substrate proteins on the outer membrane of mitochondria [5,6]. Our results show that CCCP-induced polyubiquitinated Parkin’s aggregation precipitates on both the outer and inner mitochondrial membranes non-specifically.

Although 20 mM H_2_O_2_ generated an HMW of longitudinal smear compared to CCCP and MPTP treatments, CCCP, MPTP, and H_2_O_2_ led to very similar structural and dynamic changes in the Parkin protein in WB analysis of cell cultures (Figure 2A,E and Figure 3D). Specifically, when CCCP and MPTP collapse the mitochondrial membrane potential, various ROS, including H_2_O_2_, electrolytes, and proteins, leak from the mitochondria into the cytosol or are exposed to the surface of the mitochondria, such as PINK1. As a result, CCCP creates the same environment, which is provided by exogenous H_2_O_2_ treated from the outside of the cells. Then, by exposing CCCP, MPTP, or H_2_O_2_ to the cytosol, Parkin forms self-aggregation and autoubiquitination, resulting in insolubility and the simultaneous loss of E3 ligase activity for its substrate.

It has also been found that the GFP portion of GFP-Parkin can be used as a pseudosubstrate to monitor the E3 ligase activity of Parkin [7,17]. Although GFP-Parkin cannot undergo autoubiquitination without its own E3 activity, this differs from the characteristic of ubiquitinating Parkin’s substrates. Burchell et al. (2012) [18] reported that the use of N-terminal epitope tags for the Parkin protein, especially large tags (that is, MBP and GFP), influences the physical stability and activity of Parkin, disturbs its autoinhibited native state, and results in activity for its autoubiquitination [18]. Our experiment demonstrated that compared to the HA-Parkin, the GFP-Parkin formed self-aggregation and autoubiquitination more easily, resulting in insolubility, particularly under oxidative stress (Figure 2A,C). Considering the above, we chose the FAF1 protein, one of the typical Parkin substrates, to monitor the true E3 activity of the Parkin in the cytosol. We also decided to use the untagged Parkin protein for further experiments to eliminate the effect of the Parkin tag.

In general, as multiple factors and steps are involved in an enzymatic reaction, including the polyubiquitination of substrates, it is difficult for the ubiquitination to occur in a moment compared to a chemical reaction, such as a redox reaction. The oxidation–reduction reaction of Parkin is a typical rapid chemical reaction that occurs under oxidative stress. The aggregated E3 ligase of Parkin is considered the one form of Parkin that cannot induce polyubiquitination of its substrates, which should be called the “Parkin dilemma (between E3 Parkin and redox Parkin).” In addition, even among the exogenous and hugely overexpressed Parkin proteins, almost all of them precipitate into the mitochondria as they aggregate through CCCP treatment. Thus, a very small amount of endogenous Parkin reacts with the mitochondrial H_2_O_2_ via CCCP and is thought to be completely exhausted in the cells.

Many autopsy cases of AR-JP have been reported compared to the number of autopsies of other hereditary PD cases. Importantly, the deposition of Parkin’s specific substrate or abnormal mitochondrial accumulation has thus far not been reported in the brains of patients with AR-JP in relation to a deficiency of Parkin’s E3 ligase function. In general, for cases of AR-JP pathology, neuronal loss and gliosis have been shown to be localized in the substantia nigra (SN) and locus coeruleus (LC), and they have not been accompanied by Lewy bodies or Lewy neurites [19,20,21]. Iron staining for AR-JP brain sections is more intense than that for controls and sporadic PD [22]. This suggests that oxidative stress plays a critical role in the nigral neurodegeneration of AR-JP. Nigral neurons have a melanin-poor content or immature melanin, but the mechanism of the malformation process has not yet been identified. However, a recent study showed that Parkin binds dopamine radicals via cysteine residues and enhances melanin formation in vitro, and that Parkin colocalizes with neuromelanin in the SN of AR-JP brains [10,11]. These results indicate the importance of the redox molecule Parkin in AR-JP pathology.

Selective neuronal death in the SN and LC of the AR-JP brain cannot be explained by the mitophagy deficiency hypothesis because mitochondria are distributed among all the cells in a living body. Parkin is also expressed in various tissues. In particular, it is strongly expressed in tissues that require a large amount of ATP and produce a large amount of ROS, such as skeletal muscle and myocardium [1].

In contrast, Parkin as a redox molecule binds to dopamine metabolites and reduces their toxicity [10,11]. Furthermore, in this study, we demonstrated that Parkin reacted with and reduced H_2_O_2_ derived from monoamine oxidase in vitro, which exists specifically in the mitochondria in SN and LC, in addition to the H_2_O_2_ derived from all the mitochondrial matrices in the cells (Figure 1A1,A2).

We performed a WB analysis for the mitochondrial fraction of untagged Parkin-overexpressing cells with CCCP and H_2_O_2_ treatment compared to untreated cells. Anti-Parkin antibodies detected a HMW of high-intensity longitudinal bands in both treatments (Figure 3E–G and Figure 5B1,B2). As ROS generate Parkin’s self-aggregation and autoubiquitination, these bands indicate Parkin’s accumulation on the outer and inner mitochondrial membranes. Through a WB analysis in a cell culture system overexpressing several combinations of FLAG-PINK1/untagged Parkin/HA-Ubi, Parkin aggregates of all sizes phosphorylated by PINK1 were observed (Figure 6A,C).

Recent studies do not support the close relationship between Parkin and PINK1 in mitophagy [23]. Han et al. reported that an appropriate level of nitric oxide was sufficient to trigger the translocation of Parkin, even in the absence of PINK1 [24]. A study by McWilliams et al. indicated that a lack of PINK1 did not influence basal mitophagy, even though Parkin is activated by disrupting membrane potential depolarization [25]. They clearly showed evidence that PINK1 is detectable at a basal level, and that basal mammalian mitophagy arises in the absence of PINK1. Furthermore, some iron chelators (loss of iron) induce mitophagy, which requires cells to undergo glycolysis, but does not require the stabilization of a phosphokinase, PINK1, or the activation of E3 Parkin, and occurs in primary human fibroblasts isolated from patients with AR-JP and controls [26]. Thus, various agents or factors, including Parkin self-aggregation, facilitate mitophagy independent of PINK1 or Parkin, even in patients with AR-JP.

Finally, Parkin overexpression alone seems to generate Parkin aggregation (without polyubiquitination of Parkin itself or its substrates). Gene therapy with the overexpression of the Parkin protein, which is a strong antioxidant, is a potential cause-directed therapeutic treatment for AR-JP. However, several reports have suggested that Parkin aggregation is insoluble and toxic to cells [27,28]. Therefore, further studies are required to determine whether Parkin gene therapy represents a worthwhile remedy. In contrast, glutathione (GSH) may attract attention once more, although GSH therapy has not advanced beyond the status of alternative medicine. GSH is a tripeptide comprising glutamic acid, cysteine, and glycine. The thiol group of the cysteine residue eliminates peroxide and ROS, which is the same mechanism used to remove ROS by the Parkin protein. The safety of GSH or N-acetylcysteine (NAC, a precursor of GSH) has already been established; it has been utilized as an antidote by intravenous feeding for aspirin poisoning or preeclampsia. For a practical and useful GSH or NAC treatment method, it is necessary to administer them sustainably and deliver them to the mitochondria with high efficiency.

## 4. Materials and Methods

### 4.1. In Vitro Fas Associated Factor 1 (FAF1) Polyubiquitination Assay by Parkin

The polyubiquitination assay was performed as previously described by Kitada et al. [9]. Briefly, a 100 μL reaction mixture including E1 enzyme 100 nM (Boston Biochem, Minneapolis, MN, USA, Cat#E-304-050), UbcH7 5 µM (Boston Biochem, Minneapolis, MN, USA, Cat# E2-640-100), recombinant human Parkin 1.5 µM (Boston Biochem, Minneapolis, MN, USA, Cat#E3-160-025), recombinant human phospho-ubiquitin (S65) (Boston Biochem, Minneapolis, MN, USA, Cat# U-102), and final of 1× E3 ligase buffer (Boston Biochem, Minneapolis, MN, USA, Cat# B-71). FLAG-FAF1 protein purified through immunoprecipitation was added at 3 different ratios (Parkin: FAF1 1:0, 1:2, 1:3, 1:6). The samples were incubated for 60 min at 37 °C, and the reaction was terminated by adding 1× loading buffer, and samples were separated using SDS gel for the Western blot (WB) analysis using anti-Parkin, anti-FLAG, and anti-Ubi antibodies following standard protocol. All the reagents and antibodies used in the experiment are listed in Table 1, Table 2 and Table 3.

### 4.2. The Monoamine Oxidase (MAO) Assay

The MAO activity assay was performed using a Monoamine Oxidase assay kit (Abcam, Boston, MA, USA, Cat# ab241031; Table 2) according to the manufacturer’s instructions. Briefly, the reaction mix was prepared by adding 47 µL of assay buffer, 1 µL developer, 1 µL MAO substrate and 1 µL probe; the background reaction mix was prepared by adding 48 µL assay buffer, 1 µL developer and 1 µL probe; and the enzyme solution contains 49 µL assay buffer and 1 µL MAO-A enzyme. 50 µL of reaction mix was added to 50 µL of either enzyme solution or background solution in a 96-well plate. The plate then incubated for 20 min at room temperature before adding a final concentration of 25 and 50 nM of Parkin or assay buffer for control (10 µL) for 3 min, measuring the fluorescence (Ex/Em 535/587) for 0, 10 and 20 min (readings were taken using a microplate reader Infinite M200PRO-TECAN, Männedorf, Switzerland). The background of the Parkin protein alone without the reaction was subtracted from the reaction samples. The total amount of H_2_O_2_ generated from the reaction (pmol) was calculated according to the incubation time.

### 4.3. Cell Culture, Treatment and Antibodies

The human embryonic kidney (HEK) cells obtained from ATCC. were routinely cultured in Dulbecco’s MEM/Glutamax media (Gibco BRL, Rockville, MD, USA), containing 10% fetal bovine serum and 1% penicillin–streptomycin (P/S; 100 U/mL penicillin, 100 mg/mL streptomycin, Gibco). The CCCP and H_2_O_2_ were used to treat the cells at different concentrations and times, as mentioned below. The transfection of cells with different plasmids was performed using the transfection reagent kit Lipofectamine 3000 according to manufacturer protocol (ThermoFisher Scientific, Waltham, MA, USA).

### 4.4. Cells Treatment and Preparing Cell Lysate

Transfected or control cells treated with CCCP (20 µM for 6 h) or H_2_O_2_ (20 mM for 30 min) or at different concentrations, as indicated in the results section, were lysed as soluble and insoluble fraction by 0.1% triton-X 100 (Sigma-Aldrich, St. Louis, MO, USA). In brief, soluble and insoluble fractions were isolated from a cell lysate by adding 0.1% Triton X-100 to lyse the cell pellets. The resulting lysate was centrifuged at 14K rpm at 4 °C for 10 min to remove the large cellular debris. The supernatant, which contained the Triton X-100 soluble fraction, was carefully collected and transferred to a new tube. The protein concentration of the soluble fraction was then measured using the BCA protein assay kit. To separate the insoluble fractions, the remaining pellets were sonicated in non-denaturing 1× loading buffer (without reducing agents). The resulting samples were loaded directly into the SDS gel, with precautions taken to avoid boiling the samples. For the whole cell lysate (WCL), cells were lysed with 1× RIPA buffer (Millipore, Burlington, MA, USA). The total protein for the WCL and soluble fraction was estimated using a BCA assay (ThermoFisher Scientific, Waltham, MA, USA) prior to loading in SDS-PAGE for a Western blot analysis.

### 4.5. Fluorescent Immunocytochemistry (ICC)

The human embryonic kidney (HEK) cells were seeded (50K cells/well) on 13-mm poly lysine-treated coverslips in a 4-well plate for 24 h. Seeded cells were then transfected to overexpress GFP-Parkin, HA-Ubiquitin, and Myc-Pink1 **(**0.5 µg for each**)** or GFP-Parkin and HA-ubiquitin (0.5 µg for each**)** using the transfection reagent kit Lipofectamine 3000 according to manufacturer’s protocol (ThermoFisher Scientific, Waltham, MA, USA). After 24 h of transfection, cells were treated with 20µM CCCP for 5 min or 6 h.

Following the CCCP treatment, the cells were washed with PBS, then fixed with 4% Paraformaldehyde in PBS for 10 min. Fixed cells were permeabilized and then blocked with 3% BSA (A9418, Sigma) in PBS-T (1:2000) for 1 h at room temperature. Double stain was achieved through overnight incubation with the primary antibodies in the blocking solution at 4 °C. The primary antibodies used were HA-tag (CST; 2367) (1:100) to stain Ubiquitin; COXIV (CST; 4850) (1:200); and TOM20 (Santa Cruz; sc-11415) (1:200). After their washing, the cells were incubated for 1 h at room temperature with the appropriate secondary antibodies. The secondary antibodies were Goat anti-mouse Alexa Fluor (Invitrogen; 594 A-11032,) (1:500) and Goat anti-rabbit Alexa Fluor 405 (Abcam; ab175652) (1:200). A Fluoroshield (Sigma; F6182) was used for their mounting. Images were visualized and captured using a Leica DFC 3000 G microscope by Leica Application Suite X (2.0.0.14332) software at 100× magnification. We have performed the analysis to show the Parkin translocation to the mitochondria using Image J 1.53K software. Briefly, cells were randomly selected in both the CCCP-treated group and the untreated group, and the overlap of Parkin and mitochondrial markers per cell was assessed. More than 25 cells in each group were analyzed, and Pearson’s correlation coefficient value was generated using JACoP plugin (ImageJ) [29]. Pearson’s correlation values were used to perform statistical analysis (*t*-test by Prism 5 GraphPad software) and to construct the graph.

### 4.6. Mitochondria and Cytosolic Fraction Isolation and Proteinase K (PK) Digestion

The mitochondrial and cytosolic fractions for the cells were carried out using a mitochondria isolation kit for cultured cells (ThermoFisher Scientific, Waltham, MA, USA). Following manufacturer’s guidelines, the pelleted cells were resuspended in 800 µL reagent A, a vortex for 5 s at medium speed, and then incubated for 2 min on ice; then, 10 µL of reagent B was added and vortexed at maximum for 5 s, vertexing every minute while the samples were incubated on ice for 5 min; after adding 800 µL reagent C, the tubes are mixed by being inverted several times and then centrifuged at 700× *g* for 10 min at 4 °C, the supernatant was transferred to new tube and centrifuged at 3000× *g* for 15 min at 4 °C. The supernatant represents the cytosolic fraction that was transferred to a new tube, while the pellets represent the mitochondrial fraction that was washed once by reagent C and saved for further experiments. For PK digestion, the mitochondria fraction was resuspended with isotonic solution (0.28 M sucrose, 20 mM HEPES, pH 7.5), and a final concentration of 5 µg/mL PK was added. The samples were incubated at 25 °C for different incubation times. The digestion reaction was then terminated by adding 2 mM PMSF.

### 4.7. In Vivo MPTP Injection

Male, 8–10-week-old C57BALB/6 mice were bred in the animal research facility of the College of Medicine and Health Sciences (United Arab Emirates University, UAE) and used for in vivo experiment. The study was approved by UAE University Animal Ethics committee (ERA-2018-5824). We confirm that all the methods were performed in accordance with the relevant guidelines, regulations, and ARRIVE guidelines. The mice were divided into three groups: The MPTP exposure group (Group 1), the MPTP + GSH group (Group 2) and the GSH-only group (Group 3). The mice were challenged with MPTP, MPTP + GSH or GSH only with four doses every 2-h in a single day (16 mg/kg, intraperitoneal, measured as free base; MPTP-HCl; Sigma-Aldrich and 20; GSH; 20 mg/kg·b·wt). The mice were anesthetized with a ketamine/xylazine cocktail solution (ketamine 100 mg/kg body weight and xylazine 20 mg/kg body weight) 1.5 h after the last injection, and a cardiac perfusion was performed using saline to wash out the blood. The brains were quickly removed and placed on a brain matrix (Rodent Brain Matrix, ASI instrument, MI, USA). Striatum and midbrain tissue was collected quickly and snap frozen in liquid nitrogen. The samples were processed as Triton soluble and insoluble fraction. A Western blot analysis was carried out using the samples.

### 4.8. SDS-PAGE and Immunoblotting

The total proteins were estimated and quantified using the BCA method (ThermoFisher Scientific, Waltham, MA, USA). The reduced protein samples (containing 1× reduced loading buffer and boiled for 10 min) or non-reduced samples (containing 1× non-reduced loading buffer without boiling) were separated by SDS-PAGE and transferred to PVDF membranes using an electrophoretic transfer system (Bio-Rad, Hercules, CA, USA). The membranes were then blocked with 5% non-fat dry milk in 1× PBS containing 0.1% Tween 20 (PBS-T) for 1 h, followed by incubation with primary antibody against targeted proteins as shown in Table 3. The membranes were then incubated with the corresponding secondary antibody at room temperature for 1 h. The protein recognized by the antibody was visualized using a West Pico plus chemiluminescence kit (ThermoFisher Scientific, Waltham, MA, USA). The blots were stripped and re-probed for GAPDH as a loading control.

## Figures and Tables

**Table 1:** List of plasmids.

Plasmid DNA	Source	Catalogue
pRK5-HA-Parkin	Addgene, MA, USA	17613
pCDNA3-Flag-FAF1	Genscript, NJ, USA	Custome designe
pRK5-HA-Ubi	Addgene, MA, USA	17608
pEGFP-Parkin	Addgene, MA, USA	45875
pCMV3-non-tagged Parkin	Sino Biological, Beijing, PR China	HG12092-UT
pAdtrack-CMV3-FLAG-PINK1 FL	Haque et al.	PNAS, 5 February 2008; 105 (5): 1716–1721. ??

**Table 2:** List of chemicals.

Chemical	Source	Catalogue
proteinase K	Sigma-Aldrich-USA	
MOA kit	Abcam-USA	Ab241031
Mitochondria isolation Kit for mammalian Cells	ThermoFisher Scientific-USA	89874
SuperSignal West Pico Plus Chemiluminescent Substrate	ThermoFisher Scientific-USA	34580
Pierce BCA protein Assay Kit	ThermoFisher Scientific-USA	23225
UltraCruz Autoradiography film	Santa Cruz-USA	SC-201697
Recombinant Human His6-Ubiquitin E1 Enzyme (UBE1), CF	Boston Biochem-USA	E-304-050
Recombinant Human Parkin pS65, CF	Boston Biochem-USA	E3-166-025
Recombinant Human Ubiquitin Biotin Protein, CF	Boston Biochem-USA	UB-570-100
MgATP Solution	Boston Biochem_USA	B-20
Recombinant Human Parkin, CF	Boston BiochemUSA	E3-160-025
Recombinant Human UbcH7/UBE2L3, CF	Boston Biochem-USA	E2-640-100
Recombinant Human Ubiquitin	Boston BiochemUSA	U-100H
10X E3 Ligase buffer	Boston BiochemUSA	B-71
Recombinant Human Phospho-Ubiquitin (S65) Protein, CF	Boston BiochemUSA	U-102
Carbonyl cyanide 3-chlorophenylhydrazone “CCCP”	Abcam-USA	ab14122

**Table 3:** List of antibodies.

Antibody	Host	Source	Catalogue
Parkin	Mouse	Santa Cruz-USA	SC-32282
Flag	Rabbit	Cell Signaling Technologies-USA	2368S
GAPDH	Rabbit	Cell Signaling Technologies-USA	2118S
HA	Mouse	Cell Signaling Technologies-USA	2367S
Ubiquitin	Mouse	Cell Signaling Technologies-USA	3936S
Ubiquitin	Mouse	Boston Biochem-USA	A-104
LC3	Rabbit	Cell Signaling Technologies-USA	12741S
COX IV	Mouse	Abcam-USA	ab33985
TOM 20	Rabbit	Santa Cruz-USA	Sc-11415
TH	Mouse	Immunostar-USA	22941

## Data Availability

The datasets used and analyzed during the current study are available from the corresponding author upon reasonable request.

## Supplementary Materials

The supporting information can be downloaded at: https://www.mdpi.com/article/10.3390/ijms24109027/s1.
