# Supplementary material for: Parkin Precipitates on Mitochondria via Aggregation and Autoubiquitination
Source: Int J Mol Sci. 2023 May 19;24(10):9027. doi: 10.3390/ijms24109027 (PMC10219150; doi:10.3390/ijms24109027)
Supplement: Supplementary file 1 [file ijms-24-09027-s001.zip › ijms-2367861-supplementary.pdf]

Supplementary Figure 1

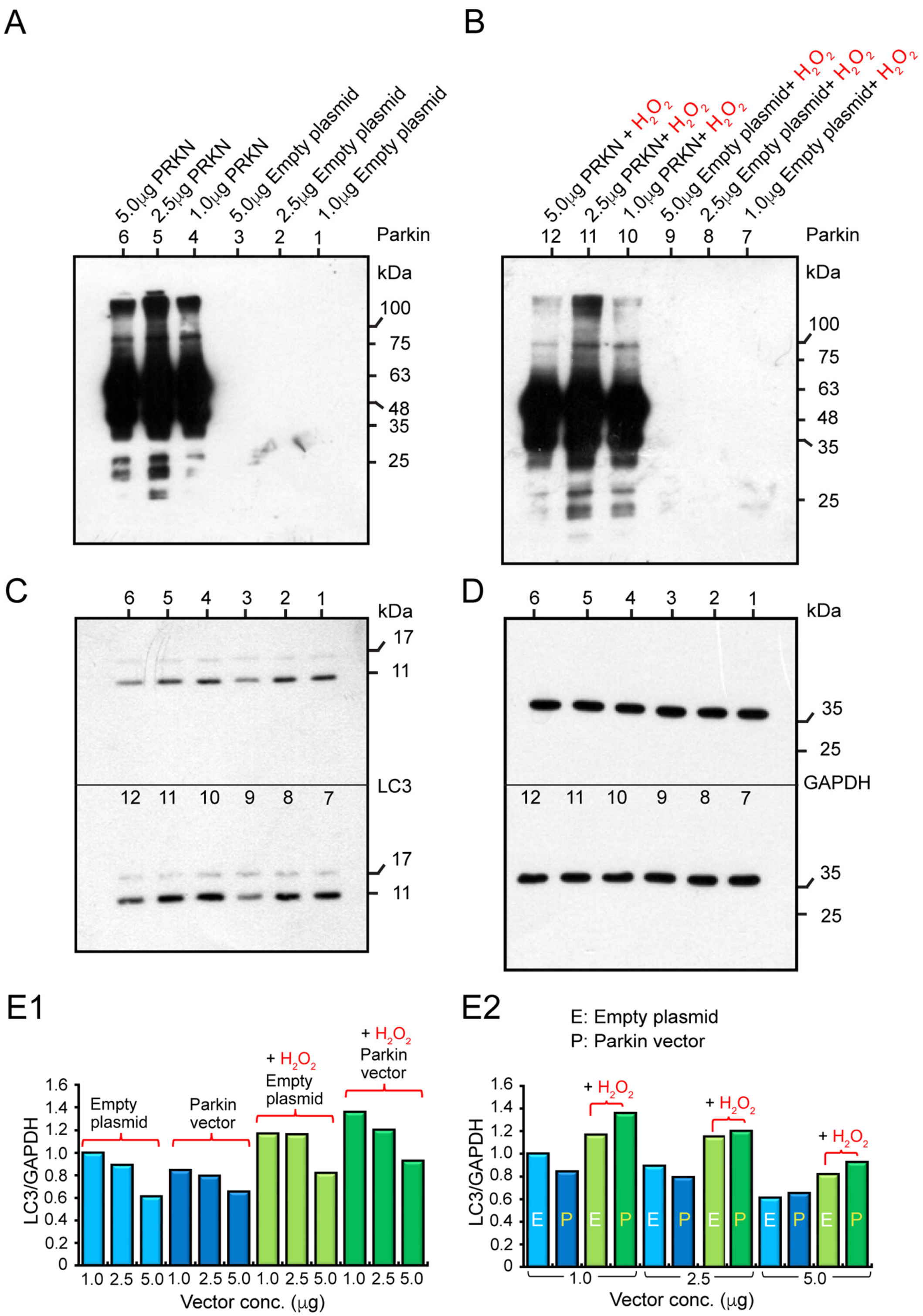

Supplementary Figure S1: The experiment followed the method shown in Figure 3B. Three doses of the Parkin gene and empty plasmid vectors were transfected into the cells. The cells were then treated with H_2O_2 or without H_2O_2, collected, and cell lysates were prepared for Western blot analysis. Parkin-transfected and empty plasmid vector-transfected cells treated with or without H_2O_2 were loaded onto two separate gels respectively. They were run together to minimizes any artifact (**A**,**B**). The transfer, development, and exposure were performed at the same time, still minimizing artifacts (**C**,**D**). The ratio of LC3 to GAPDH of empty vector group (without H_2O_2 treatment) was set to 1.0 as a control. Values of other groups are calculated with reference to the above control.

Result: As shown in (**E1**), we compared LC3/GAPDH in four groups, divided by the two kinds of vectors in the presence or absence of treatment with 1 mM of H_2O_2. Although LC3 was primarily enhanced by H_2O_2, its expression was inversely proportional to the concentration of Parkin (1.0 μg > 2.5 μg > 5.0 μg), perhaps due to Parkin's reacting with and reducing H_2O_2. However, because a similar trend was observed with the overexpression of empty vectors, we re-evaluated those LC3 expressions in three groups according to the amount of empty or Parkin gene vectors (**E2**). As Suppl-Figure 1E2 shows, treatment with 1 mM of H_2O_2 also enhanced LC3's expression; however, when treated with H_2O_2, overexpression of the Parkin gene vector further intensified LC3's expression at every vector concentration. Thus, a simple aggregate of Parkin modulates seemed to increase LC3's expression.

Supplementary Figure 2

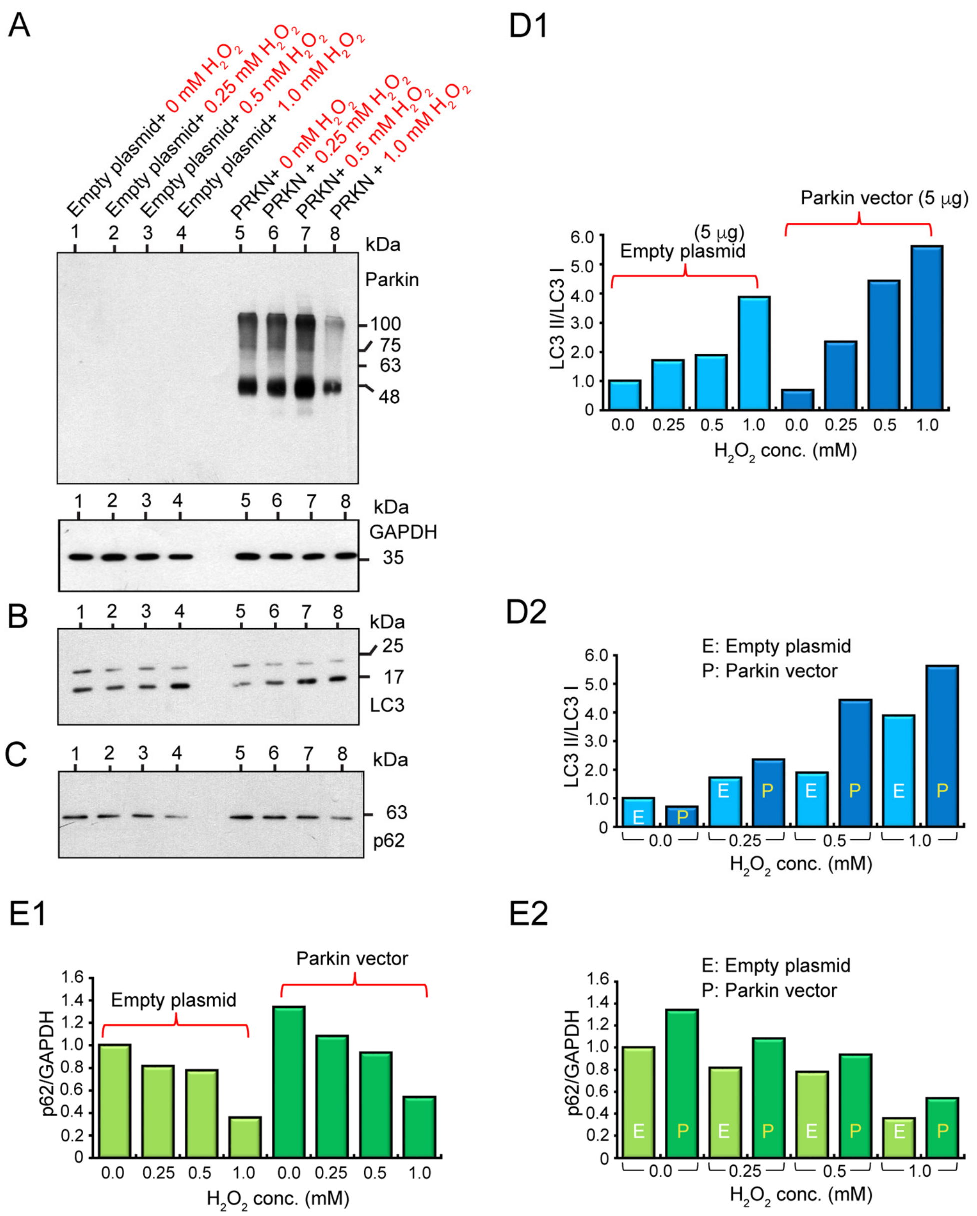

Supplementary Figure S2: This experiment was conducted in the same manner as the one in Suppl-Figure 1. The only difference is that this time we did not change the concentration (=5 μg alone) of Parkin vector and control empty plasmid but treated the cultured cells with different concentrations of H_2O_2 (0, 0.25, 0.5, and 1.0 mM) and performed WB analysis for LC3 and p62, which are key molecules involved in autophagy (**2A–C**). The data (LC3 II/I and p62/GAPDH) is standardized as 1.0 for LC3 II/I or p62/GAPDH of 0 mM H_2O_2 treated cells transfected with 5μg empty plasmid.

Result: Both empty plasmid and Parkin vector overexpression increased LC3 activity (LC3 II/I) in an H2O2 concentration-dependent manner (**D1**). However, when comparing the two groups in terms of individual H_2O_2 concentrations, LC3 activity was higher in the Parkin vector overexpression group, even though Parkin originally diminishes H_2O_2 concentration (**D2**). Thus, as discussed in Suppl-Figure 1, it is possible that Parkin aggregation positively modulates LC3 activity.

A receptor protein that recognizes ubiquitin chains and directs specific proteins and cell organelles to autophagy, p62/SQSTM1 is degraded in an autophagy-dependent manner together with autophagy cargo. In both empty plasmid and Parkin vector overexpression in cells, p62 decreases inversely proportional to H_2O_2 concentration (**E1**). At the same H_2O_2 concentration, empty plasmid overexpression had less p62 than Parkin vector overexpression (**E2**). It is considered that Parkin reacts with H_2O_2, resulting in a decrease in the amount of H_2O_2, which induces a decrease in autophagy activity and p62 degradation. In other words, higher concentrations of H_2O_2 promote autophagy and p62 is more degraded. Thus, the results of this experiment also support the experimental results in Suppl-Figure 1.

Supplementary Figure 3

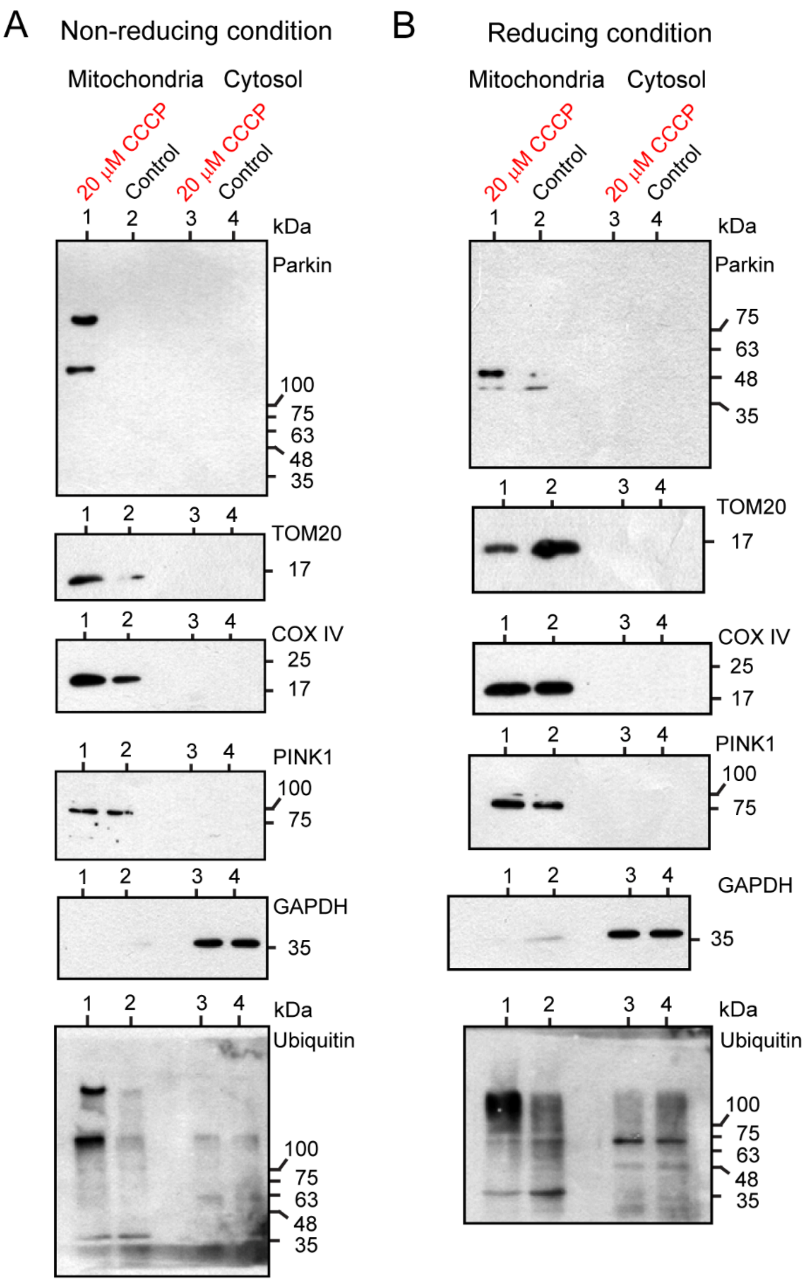

Supplementary Figure S3: Accumulation of endogenous Parkin in mitochondria under CCCP exposure of SH-SY5Y cells. SH-SY5Y cells were cultured for 24 hr and then harvested 6 hr after treatment with CCCP (20 ◎M). WB analysis of mitochondrial fractions was performed on the resulting cell lysates under reducing or non-reducing conditions using antibodies against Parkin, PINK1, ubiquitin, TOM20 and COX-IV (Suppl-Figure 3).

Result: We confirmed that endogenous Parkin is detectable in the SH-SY5Y cell line, which is a dopamine-producing and has MAO A/B on mitochondria. SH-SY5Y cells were cultured for 24 hours and then treated with CCCP (20 μM) for 6 hours. Cells were then harvested and processed to obtain mitochondria and cytosolic fractions. Western blot (WB) analysis was carried out under non-reducing conditions using anti-Parkin antibodies revealed two bands of dense high-molecular-weight aggregates of endogenous Parkin (**A**). Moreover, the result of WB using anti-ubiquitin (Ubi) antibodies showed that these aggregates are ubiquitin positive.

By contrast, under reducing conditions, a band of Parkin monomer was detected in WB with anti-Parkin antibodies (**B**). In WB analysis using anti-Ubi antibodies, vertical smears were observed below the molecular size of these aggregates, apparently forming polyubiquitination of mitochondrial proteins by Parkin monomer with E3 activity. However, when compared with the results of WB under non-reducing conditions using the same specimens, it is clear that presence of DTT in the loading dye degrades these high-molecular-weight Parkin aggregates to various sizes, which are ubiquitin positive. Thus, the results of a series of experiments using a multi-gene expression system were consistent with the results of similar experiments using endogenous Parkin in SH-SY5Y cells.
